# Predicting Sprint Potential: A Machine Learning Model Based on Blood Metabolite Profiles in Young Male Athletes

**DOI:** 10.1002/ejsc.12272

**Published:** 2025-02-24

**Authors:** Jingfeng Chen, Yuhang Qian, Yuansheng Xu

**Affiliations:** ^1^ School of Sport, Exercise and Health Sciences Loughborough University Loughborough UK; ^2^ School of Architecture, Building and Civil Engineering Loughborough University Loughborough UK

**Keywords:** athletic potential, biomarkers, machine learning, metabolites

## Abstract

This study aims to utilize male blood metabolite signatures for (i) distinguishing between healthy individuals and athletes, thereby optimizing the athlete screening process; and (ii) predicting athletic performance in 100, 200, and 400 m sprints, enhancing precompetition preparation and intervention strategies. Initially, we employed nontargeted metabolomics to analyze the blood metabolome of healthy individuals (*n* = 10) and athletes (*n* = 10), identifying differential expressed metabolites (DEMs) potentially related to athletic performance through differential analysis, consensus clustering, WGCNA, and UMAP analysis. Subsequently, using LASSO‐Cox analysis, we refined our selection to two core DEMs: HMDB0012085 (Sphingomyelin (d18:0/14:0)) and HMDB0009224 (Phosphatidylethanolamine(20:0/18:1(9Z))) associated with athletic performance. We then applied targeted metabolomics to measure the levels of these DEMs in a larger cohort, including healthy individuals (*n* = 50) and athletes (*n* = 100), revealing a significant increase in the levels of HMDB0012085 and HMDB0009224 in athletes compared to healthy individuals. Utilizing 13 machine learning classification methods, we demonstrated that the levels of HMDB0012085 and HMDB0009224 in blood effectively differentiate between healthy individuals and athletes. Notably, HMDB0012085 exhibits greater feature importance across multiple algorithms compared to HMDB0009224. Specifically, in decision trees (94.1 vs. 5.9), random forests (60.7 vs. 39.3), gradient boosting trees (91.5 vs. 8.5), CatBoost (61.7 vs. 38.3), ExtraTrees (64.7 vs. 35.3), and XGBoost (74.5 vs. 25.5). Finally, we found a significant negative correlation between the levels of HMDB0012085 and HMDB0009224 in whole blood and sprint times for 100, 200, and 400 m races. In conclusion, HMDB0012085 and HMDB0009224 in whole blood hold promise as biomarkers for predicting athletic potential in males.

**Summary**

- Young male athletes exhibit a notably higher concentration of the metabolites HMDB0012085 and HMDB0009224 in their whole blood compared to their healthy counterparts.
- The concentration of the metabolites HMDB0012085 and HMDB0009224 in whole blood serves as a reliable indicator to differentiate between healthy individuals and young male athletes.
- The concentration of the metabolites HMDB0012085 and HMDB0009224 in whole blood is a good predictor of superior sprint performance among young male athletes.

## 1 Introduction

The athlete selection process is a holistic and detailed procedure that integrates a broad spectrum of evaluations such as physical fitness assessments, skill proficiency evaluations, physiological indicators, psychological profiling, scrutiny of athletic backgrounds, health examinations, genetic screenings, assessments of psychological fortitude, appraisals of technical and tactical acumen, assessments of adaptability to different environments, and evaluations of interpersonal and communication skills (James, Jones, and Farra 2022; Jäger et al. 2019; Bonney, Larkin, and Ball 2022; Semenova, Hall, and Ahmetov 2023; Fu et al. 2022). This multidimensional strategy is designed to pinpoint and recruit athletes who exhibit optimal potential, competencies, and psychological attributes, guaranteeing their exceptional performance in their chosen sports disciplines and their capacity to thrive across diverse training and competitive settings.

In the realm of sports, the endurance and performance of athletes are frequently evaluated through the detection of certain biological markers. Lactic acid stands out as one of the most recognized indicators. During intense endurance activities, it accumulates in the muscles, with its concentration indicating the athletes' stamina and the level of muscular exhaustion (Lee et al. 2023). Beyond lactic acid, several other markers provide valuable insights into endurance capabilities. Amino acids, the fundamental building blocks of proteins, are particularly significant. Their metabolic pathways during physical activity offer insights into muscle damage and repair processes which are essential for gauging an athlete's recovery capacity and overall performance (Ferrando et al. 2023). Oxidative stress markers also play a crucial role in assessing athletic performance. These indicators reflect the body's internal oxidative stress levels during exercise, providing a measure of an athlete's fatigue resistance and their ability to recuperate (Clarke et al. 2020). Understanding and monitoring these markers can lead to more informed training regimens and enhanced performance strategies for athletes.

The advent of machine learning in sports signifies the onset of a transformative age, serving as an integral branch of artificial intelligence (Reis et al. 2024). It employs sophisticated algorithms and statistical models to dissect intricate datasets, uncovering hidden patterns and trends. This technological integration has shifted the analytical approach in sports from a retrospective to a forward‐looking perspective that is both predictive and prescriptive. The capability to analyze extensive datasets is amplified, capturing the minute details of athletes' biomechanics and the discrete events recorded by high‐fidelity sensors. Within the sphere of training and performance enhancement, machine learning algorithms develop customized regimens by synthesizing an athlete's physiological data, competitive history, and genetic profile (Taber et al. 2024). These individualized plans are designed to fine‐tune training intensities, reduce the risks associated with excessive training, and boost the effectiveness of skill development. Moreover, machine learning is a vanguard in preventing injuries, serving as a watchful guardian against health hazards that athletes might face. It leverages real‐time data from wearable devices to spot indicators of fatigue or injury, enabling proactive measures that safeguard athletes' health and extend their professional lifespan (Amendolara et al. 2023). The aim of our research is to utilize machine learning for the discovery of blood‐based metabolic markers capable of identifying athletes with innate potential and forecasting their athletic prowess.

## 2 Materials and Methods

### 2.1 Study Design

**FIGURE 1:**
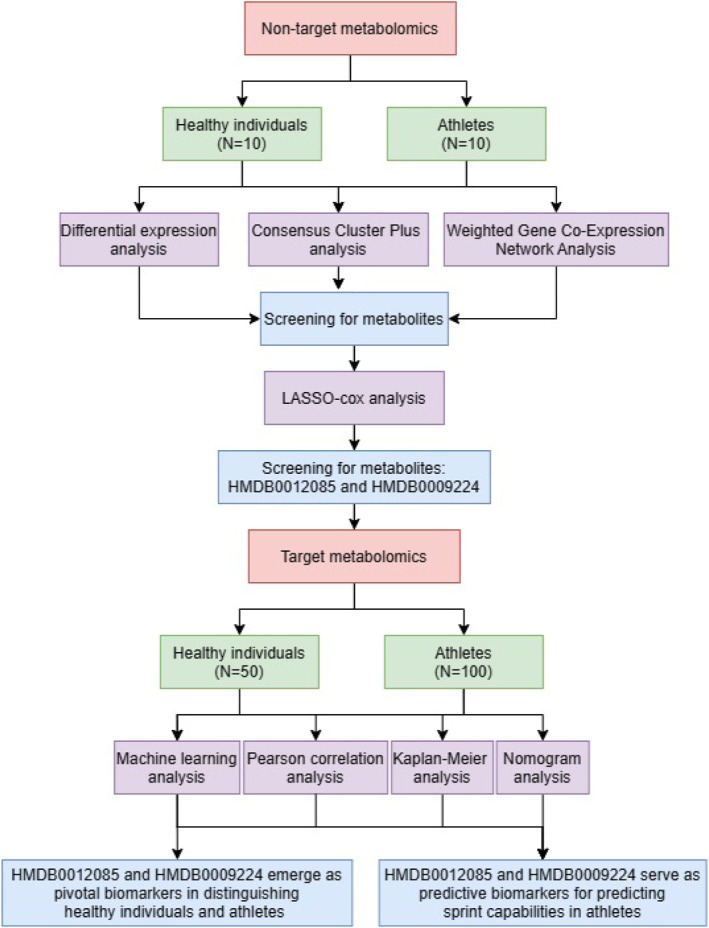
The flow chart of this study. Initially, we performed a thorough metabolite profiling analysis using nontargeted metabolomics on both healthy subjects and athletes. This was followed by a series of analytical methods, including differential expression analysis, ConsensusClusterPlus, UMAP for dimensionality reduction, and WGCNA for network‐based analysis, to preliminarily identify metabolites associated with athletic capabilities. LASSO‐Cox analysis was then utilized to fine‐tune our findings, leading to the identification of two key metabolites, HMDB0012085 and HMDB0009224. Through targeted metabolomics, we quantified their levels in the whole blood samples of both cohorts. Subsequently, we employed a suite of statistical approaches—machine learning, Pearson correlation analysis, Kaplan–Meier survival analysis, and Nomogram analysis—to assess the metabolites' ability to distinguish between healthy individuals and athletes and to predict performance metrics in athletes.

Firstly, we conducted a comprehensive blood metabolite expression analysis using nontargeted metabolomics in healthy subjects and athletes. We then applied differential expression analysis, ConsensusClusterPlus, uniform manifold approximation and orojection (UMAP), and weighted gene coexpression network analysis (WGCNA) methods to initially identify metabolites potentially linked to athletic performance. Subsequent fine‐tuning with LASSO‐Cox analysis pinpointed two pivotal metabolites: HMDB0012085 (Sphingomyelin (d18:0/14:0)) and HMDB0009224 (Phosphatidylethanolamine(20:0/18:1(9Z))). With targeted metabolomics, we quantified the levels of these metabolites in the whole blood of both healthy individuals and athletes. Employing machine learning, Pearson correlation analysis, Kaplan–Meier survival analysis, and Nomogram analysis, we evaluated the effectiveness of HMDB0012085 and HMDB0009224 in differentiating between the two cohorts and their predictive efficacy for athletes' performance metrics (Figure 1).

### 2.2 Metabolomics Detection

In alignment with the track and field athlete technical grading criteria, which designate athletes as those who complete a 100‐m dash in under 12.64 s, a 200‐m dash in under 25.74 s, and a 400‐m dash in under 56.64 s, we conducted a metabolomics study. Peripheral whole blood samples (1 mL) were collected from a cohort of 10 nonathletes and 10 athletes presprint, specifically 30 min prior to their sprint events, for nontargeted metabolomics analysis. Furthermore, for targeted metabolomics analysis, we gathered 1 mL of peripheral blood from a larger group comprising 50 nonathletes and 100 athletes, specifically 30 min prior to their sprint events. All participants were healthy male individuals with no reported diseases. For the nonathlete group (age 20 ± 3 years, body mass 66.1 ± 5.3 kg, and stature 174.1 ± 5.2 cm) and for the athlete group (age 20 ± 3 years, body mass 64.2 ± 3.6 kg, and stature 175.3 ± 4.3 cm). The metabolomics detection were conducted at Novogene (United kingdom), employing the liquid chromatography–mass spectrometry (LC–MS) technology for both the nontargeted and targeted detection approaches. To delve into the intricate metabolic profiles extracted from our samples, we leveraged MetaboAnalyst 6.0, an advanced online platform specifically designed for metabolomics data analysis and interpretation.

### 2.3 Consensus Cluster Plus Analysis

Cluster analysis was conducted utilizing the ConsensusClusterPlus algorithm, a robust class discovery tool that incorporates confidence assessments and item tracking as introduced by Wilkerson and Hayes in 2010 (Wilkerson et al. 2010). The method employed agglomerative hierarchical clustering based on 1‐Pearson correlation distances, ensuring a robust linkage measure between data points. To enhance the reliability of the clustering results, a resampling strategy was implemented, where 80% of the samples were repeatedly drawn 10 times. The determination of the optimal cluster count was facilitated by examining the empirical cumulative distribution function plot, providing a visual assessment of the data's natural grouping tendencies.

### 2.4 UMAP Analysis

The UMAP algorithm was selected for its ability to efficiently capture the local and global structures within high‐dimensional data. Utilizing the R programming language, we implemented the “UMAP” package after appropriate data preprocessing, which included log transformation and normalization to stabilize variance across samples. We carefully determined the key UMAP parameters, specifically *n*_neighbors and min_dist, through a series of sensitivity analyses to ensure the robustness and reproducibility of our low‐dimensional embeddings. The resulting two‐dimensional UMAP representation was visualized using ggplot2, allowing us to discern distinct patterns and clusters that correlated with our samples' biological annotations. This approach facilitated a comprehensive exploration of the data, providing insights into the underlying relationships within the metabolites expression profiles of our study cohorts.

### 2.5 WGCNA Analysis

WGCNA, a systems biology approach, was employed to construct a network‐based representation of the metabolome, facilitating the discovery of metabolites modules with highly correlated expression profiles. The *R* statistical environment, equipped with the “WGCNA” package, was utilized for all computational steps, including the calculation of metabolite expression correlations, the determination of an optimal soft‐thresholding power to emphasize scale‐free topology, and the hierarchical clustering of metabolites into modules based on topological overlap. Modules were further characterized by metabolites, which served as summary statistics, and their associations with continuous and categorical traits were assessed through correlation analysis. This integrative approach allowed us to explore the complex metabolites expression landscape, revealing modules with significant relevance to our study's biological questions and hypotheses.

### 2.6 LASSO‐Cox Analysis

We incorporated the LASSO‐Cox analysis to identify a subset of metabolites that are significantly associated with sprint performance (times) in our study cohort. This analytical approach integrates the penalized Cox proportional hazards model with LASSO to enhance the prediction accuracy of survival models while performing variable selection. The LASSO‐Cox method was implemented using the R programming language, employing the “glmnet package” that enables the fitting of Cox regression models with LASSO penalties. The metabolite expression data underwent rigorous preprocessing, including standardization to ensure each metabolite had a mean of zero and a standard deviation of one, to prepare for the analysis. We selected the optimal tuning parameter of the LASSO penalty through cross‐validation, which aimed to balance the bias‐variance trade‐off and maximize the predictive accuracy of the model. The Cox regression model, with the LASSO penalty, was then applied to identify metabolites whose expression levels were significantly predictive of sprint performance as indicated by the nonzero coefficients in the penalized model.

### 2.7 Kaplan–Meier Analysis

The Kaplan–Meier method was utilized to estimate the probability of participants achieving a qualifying sprint performance. The log‐rank test was applied to statistically assess differences in the curves, which represent the time to achieve this performance standard across groups stratified by varying levels of metabolites and running times.

### 2.8 Nomogram Analysis

In this study, we utilized the *R* software package “rms” to develop a predictive nomogram integrating running time, the status of meeting running standards, and two characteristic metabolites. The Cox proportional hazards model was employed as the statistical method for the analysis. The “rms” package facilitated the transformation of the Cox model into a nomogram, a graphical tool that allows for the estimation of an individual's risk of not meeting the running standard over time.

### 2.9 Machine Learning

In our machine learning classification task, we employed a comprehensive suite of 13 algorithms to classify individuals into healthy subjects and athletes, utilizing metabolites as predictive variables. This diverse set included decision trees, random forest, AdaBoost, gradient boosting trees, CatBoost, ExtraTrees, k‐nearest neighbors, backpropagation neural networks, support vector machines, XGBoost, LightGBM, naive Bayes, and logistic regression employing the gradient descent method. Detailed parameters are shown in Table S1. For our machine learning regression analysis, we deployed a versatile array of 12 methodologies to predict running times and metabolite levels. This ensemble comprised decision trees, random dorest, AdaBoost, gradient boosting trees, CatBoost, ExtraTrees, k‐nearest neighbors, backpropagation neural networks, support vector machines, XGBoost, LightGBM, and logistic regression employing the gradient descent method.

## 3 Results

### 3.1 Investigating the Variations in Blood Metabolic Profiles Between Healthy Male Subjects and Young Male Athletes

**FIGURE 2:**
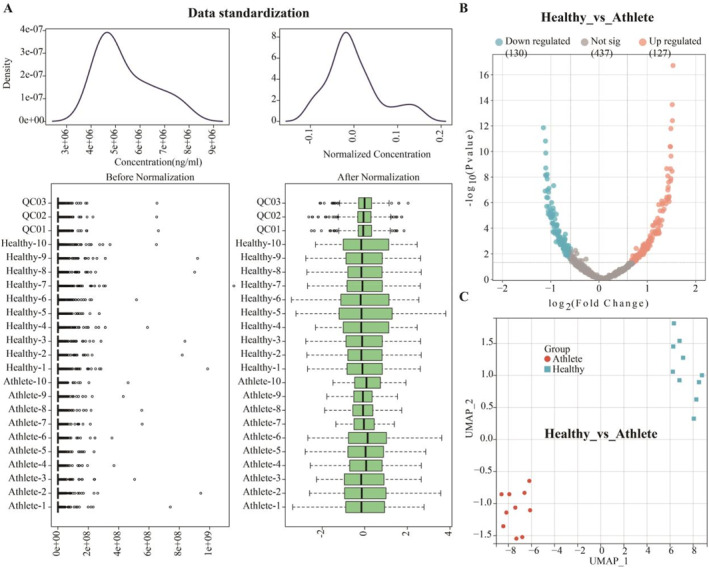
Healthy individuals and athletes display unique metabolic signatures within their whole blood profiles. (A) We normalized the data from our nontargeted metabolomics assays using the MetaboAnalyst 6.0 database, ensuring a robust statistical foundation for our subsequent analyses. (B) Applying stringent selection criteria—specifically, a |fold change| ≥ 1.5 = and a *p*‐value ≤ 0.05—we discerned a cohort of differential expressed metabolites (DEMs) in the whole blood samples, characterized as DEMs‐cohort1 which delineate the metabolic distinctions between healthy individuals and young athletes. (C) Leveraging the insights from DEMs‐cohort1, we engaged in UMAP analysis to visually and statistically contrast the metabolic variances between the healthy individuals and young athletes, revealing the underlying biosignatures that may be attributed to their physiological and athletic disparities.

Utilizing nontarget metabolomics, we explored metabolic disparities in peripheral blood samples from a cohort of healthy individuals (*n* = 10) and young athletes (*n* = 10). Initially, we subjected the metabolomics data to standard normalization procedures via the MetaboAnalyst 6.0 database (Figure 2A). Subsequently, metabolites exhibiting a |fold change| ≥ 1.5 and a *p*‐value ≤ 0.05 were identified as differential expressed between the two groups (Figure 2B). These 257 differential expressed metabolites (DEMs or DEMs‐cohort1) were then subjected to UMAP analysis, revealing distinct metabolic profiles between the healthy individuals and young athletes (Figure 2C, Supporting Information S1: Supplementary Material 1.xls). As shown in Table S2, we performed KEGG pathway enrichment analysis on the 257 DEMs using a screening threshold of *p* ≤ 0.05 via MetaboAnalyst 6.0 database. The results indicate that these DEMs are primarily enriched in the following pathways: glycerophospholipid metabolism, pentose phosphate pathway, and starch and sucrose metabolism. Additionally, we performed UMAP analysis to examine the expression patterns of all metabolites in both healthy individuals and young athletes (Figure S1).

### 3.2 Screening Metabolites Associated With Athletic Performance via Consensus Cluster Plus Analysis

**FIGURE 3:**
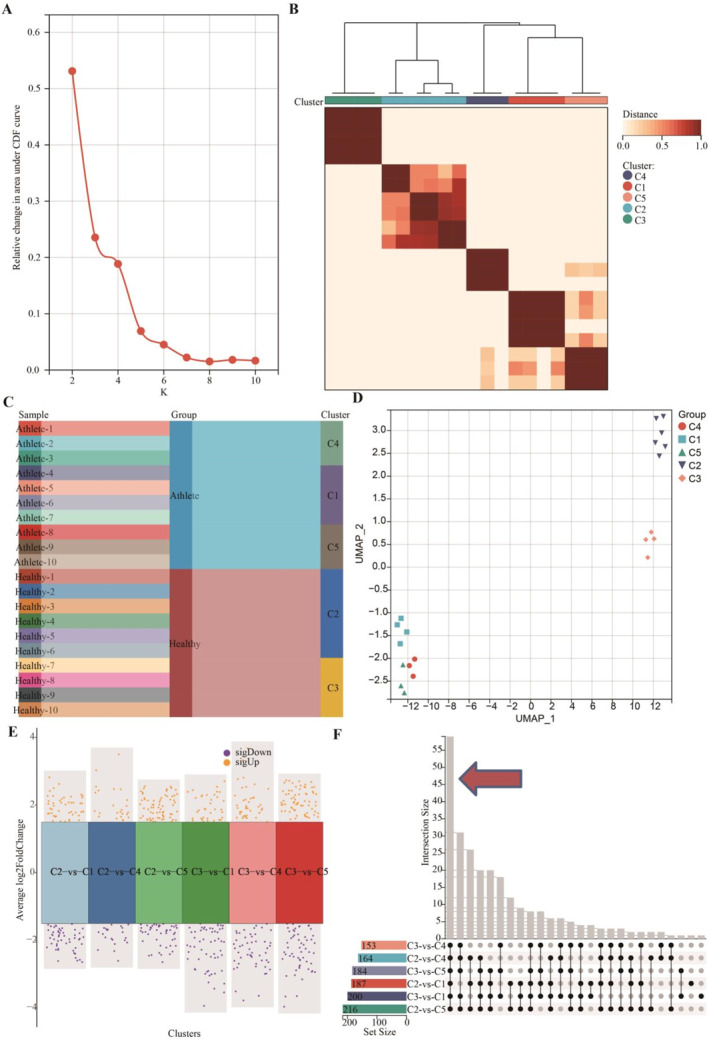
Identifying key metabolites correlated with athletic performance by leveraging ConsensusClusterPlus analysis. (A) Based on the average silhouette score of cluster consistency assessment, we set the number of clusters to *K* = 5. (B) The heatmap shows good consistency across all groups when *K* = 5. (C) Athletes are primarily categorized into clusters C1, C4, and C5, whereas healthy individuals are mainly in clusters C2 and C3. (D) UMAP analysis reveals significant differences in metabolic profiles among the five clusters. (E) Using a |fold change| ≥ 1.5 and a *p*‐value ≤ 0.05 as selection criteria, we compared the metabolic differences between clusters C1, C4, and C5 versus C2 and C3. (F) A total of 59 DEMs were identified between clusters C1, C4, and C5 and clusters C2 and C3.

We employed the ConsensusClusterPlus approach to delineate the core metabolites. Initially, leveraging the area under the distribution curve, we stratified healthy individuals (*n* = 10) and young athletes (*n* = 10) into quintiles (Figure 3A). The heatmap of the clustering outcomes demonstrated robust consistency across the clusters (Figure 3B). A sankey diagram revealed that healthy individuals predominantly fell into clusters C2 and C3, whereas young athletes were distributed across clusters C1, C4, and C5 (Figure 3C). UMAP visualization underscored variations in metabolic profiles among these clusters (Figure 3D). Utilizing stringent criteria of fold change ≥1.5 and *p* ≤ 0.05, we conducted a comparative analysis of metabolite expression between clusters C2 and C3 versus clusters C1, C4, and C5 (Figure 3E). An upset analysis highlighted 59 metabolites that were significantly differential regulated in clusters C1, C4, and C5 relative to clusters C2 and C3 (Figure 3F).

### 3.3 Screening Metabolites Associated With Athletic Performance via WGCNA Analysis

**FIGURE 4:**
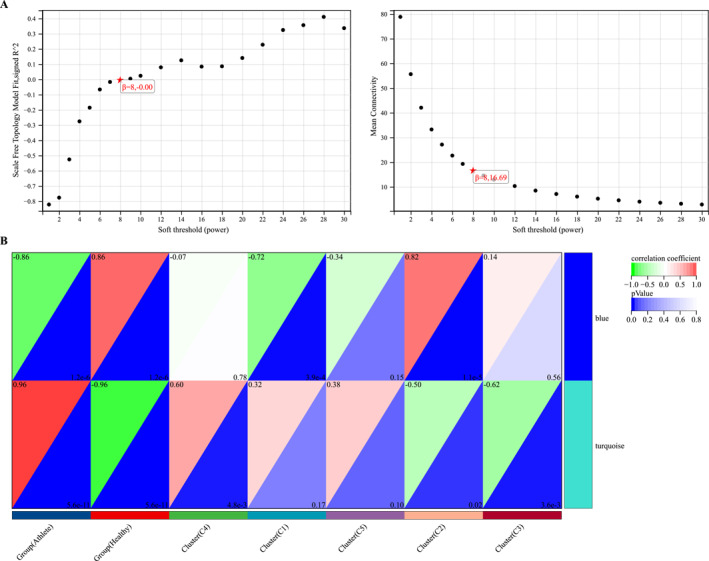
Identifying key metabolites correlated with athletic performance by leveraging WCGNA analysis. (A) Utilizing WCGNA analysis, we calculated the soft threshold for our analysis. (B) The WCGNA analysis revealed that the metabolic expression profiles are primarily divided into two modules. Pearson correlation analysis indicated that the turquoise module has a higher correlation with the grouping (athlete, healthy, and C1–C5) than the blue module.

We further utilized the WGCNA analysis to pinpoint the core DEMs within a specific subset, DEMs‐cohort1. Our approach began with an evaluation of the network's scale‐free topology and average connectivity, key parameters for network robustness and biological relevance (Figure 4A). Following this, we performed a comprehensive Pearson correlation analysis to explore the relationships between the consolidated modules and the diverse study groups (Figure 4B). Intriguingly, the correlation analysis highlighted the turquoise module—encompassing 49 DEMs—as having a significantly higher correlation with the categorized groups compared to the blue module (Figure 4B), suggesting its potential role in distinguishing the metabolic responses across groups.

### 3.4 Identifying Metabolites Serve as Metrics for Evaluating Athletic Performance in Sprint Events

**FIGURE 5:**
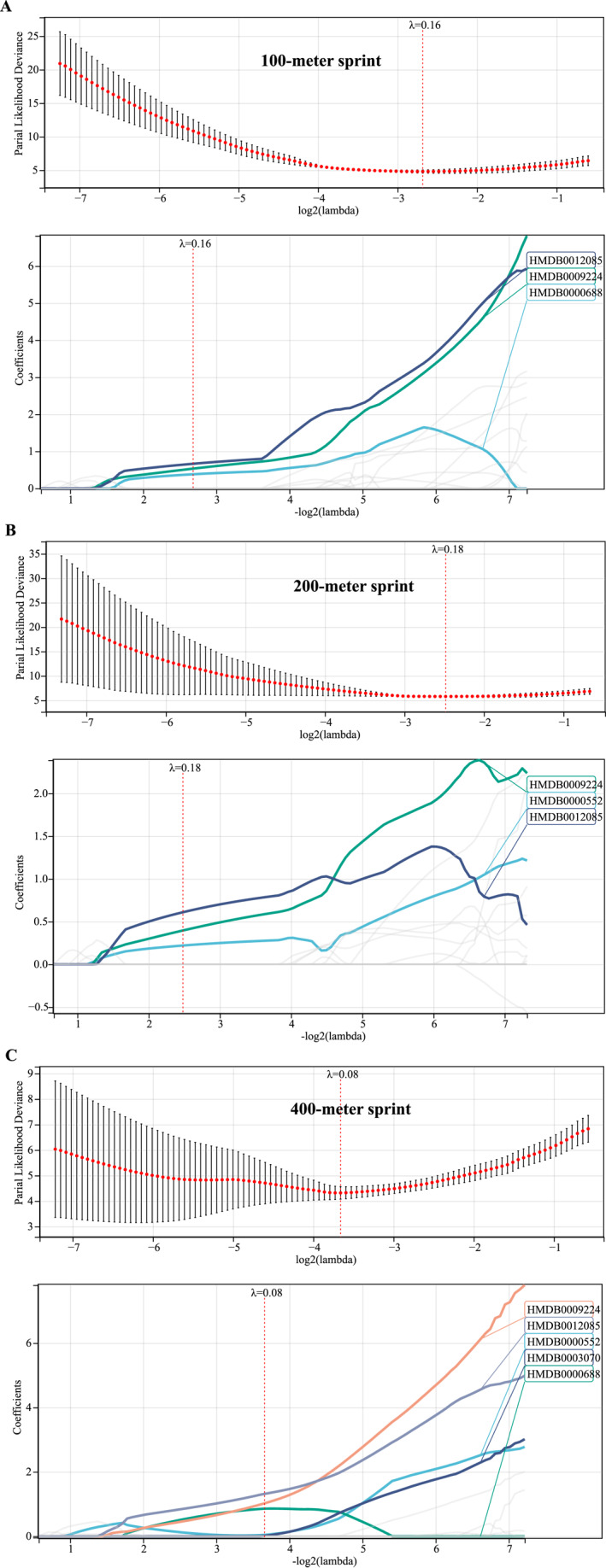
Screening core DEMs through LASSO analysis. (A) Through the LASSO regression analysis, we identified a panel of metabolites correlated with performance in the 100‐m sprint. (B) By applying LASSO regression analysis, we pinpointed specific metabolites linked to the 200‐m sprint performance. (C) With the aid of LASSO regression analysis, we selected key metabolites that are associated with performance in the 400‐m sprint.

Based on the ConsensusClusterPlus (Figure 3) and WGCNA (Figure 4) analyses, we have preliminarily identified metabolites that may be associated with athletic performance. Utilizing these metabolites, we further employed the LASSO‐Cox analysis to assess which of these are correlated with sprint performance in healthy individuals and athletes. Notably, in all three sprint distances, DEMs, HMDB0012085 and HMDB0009224, demonstrated a significant correlation with athletic performance (Figure 5A–C).

### 3.5 The Blood Metabolites HMDB0012085 and HMDB0009224 Are Effective in Distinguishing Healthy Individuals From Athletes

**FIGURE 6:**
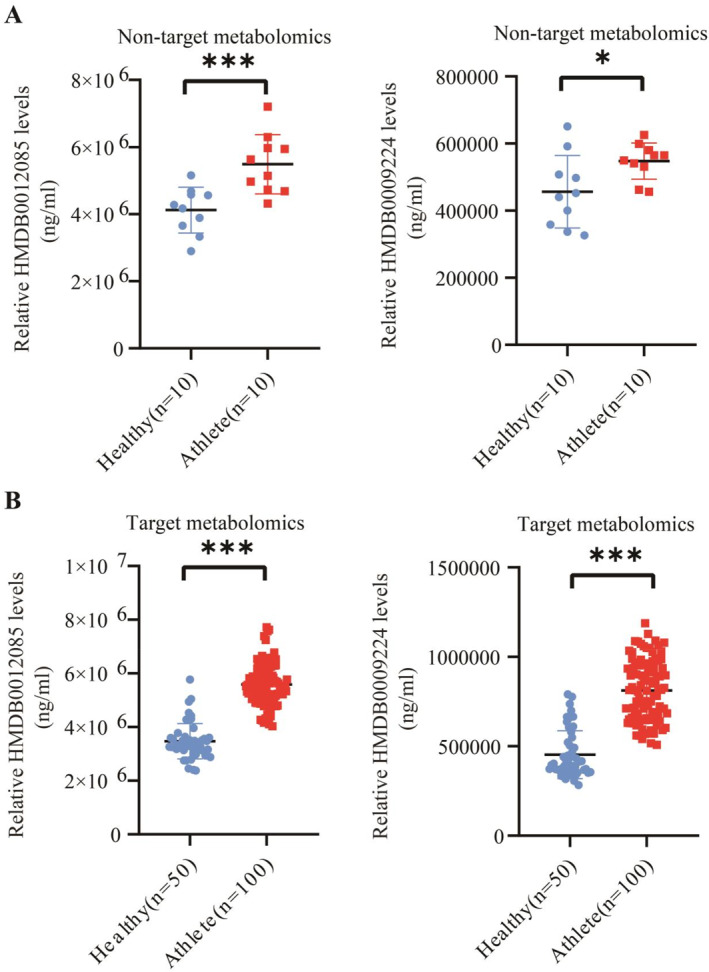
The blood metabolites HMDB0012085 and HMDB0009224 are significantly increased in athletes compared to healthy individuals. (A) HMDB0012085 and HMDB0009224 was detected in blood via nontargeted metabolomics in healthy individuals (*n* = 10) and athletes (*n* = 10). (B) HMDB0012085 and HMDB0009224 was detected in blood via targeted metabolomics in healthy individuals (*n* = 50) and athletes (*n* = 100). * represents *p* ≤ 0.05, *** represents *p* ≤ 0.001. We utilized an unpaired *t*‐test to compare the metabolite expression levels between healthy individuals and young athletes.

**FIGURE 7:**
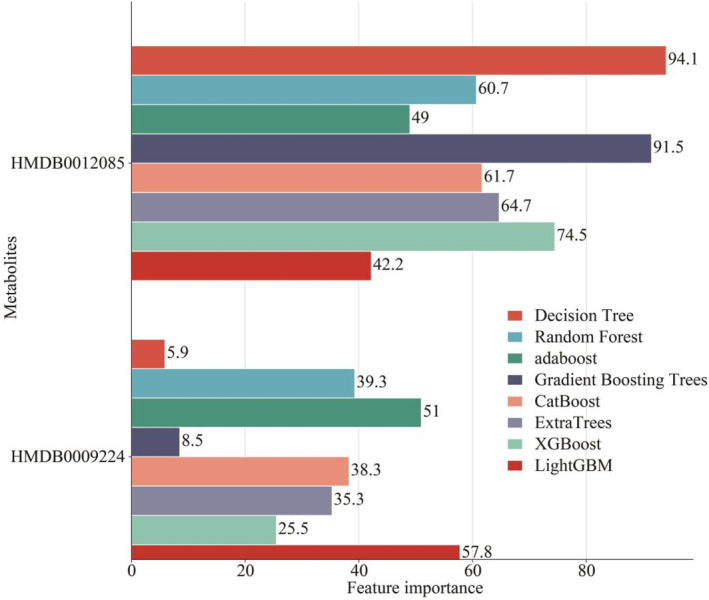
Feature importance of HMDB0012085 and HMDB0009224 in distinguishing in healthy individuals and athletes. In distinguishing between healthy individuals and young athletes, the feature importance of HMDB0012085 proved to be more significant than that of HMDB0009224.

Based on nontargeted metabolomics detection (Figure 6A) and targeted metabolomics detection (Figure 6B), we observed a significant increase in the levels of metabolites HMDB0012085 and HMDB0009224 in the blood of young athletes compared to healthy individuals via unpaired *t*‐test. Moreover, the analysis from 13 machine learning models indicates that the metabolites HMDB0012085 and HMDB0009224 are effective in distinguishing healthy individuals and young athletes (Table 1, Figure S2). In the distinguishing between healthy individuals and young athletes, the feature importance of HMDB0012085 was superior to that of HMDB0009224 (Figure 7).

**TABLE 1:** Machine learning models for blood metabolite profiling: Differentiating healthy subjects from young athletes using HMDB0012085 and HMDB0009224.

Machine learning classification methods	Set	Accuracy	Recall score	Precision	F1 score
Decision tree	Training	1	1	1	1
	Cross‐validation	0.829	0.829	0.851	0.824
	Validation	0.933	0.933	0.94	0.932
Random forest	Training	1	1	1	1
	Cross‐validation	0.886	0.886	0.903	0.884
	Validation	0.9	0.9	0.901	0.899
Adaboost	Training	1	1	1	1
	Cross‐validation	0.871	0.871	0.876	0.871
	Validation	0.9	0.9	0.903	0.901
Gradient boosting trees	Training	1	1	1	1
	Cross‐validation	0.9	0.9	0.908	0.899
	Validation	0.767	0.767	0.768	0.766
CatBoost	Training	1	1	1	1
	Cross‐validation	0.914	0.914	0.927	0.915
	Validation	0.867	0.867	0.876	0.867
ExtraTrees	Training	1	1	1	1
	Cross‐validation	0.871	0.871	0.898	0.872
	Validation	0.867	0.867	0.87	0.864
K‐nearest neighbors	Training	0.929	0.929	0.929	0.928
	Cross‐validation	0.871	0.871	0.894	0.871
	Validation	0.9	0.9	0.9	0.899
Backpropagation neural network	Training	0.529	0.529	0.279	0.366
	Cross‐validation	0.443	0.443	0.213	0.284
	Validation	0.433	0.433	0.188	0.262
Support vector machine	Training	0.743	0.743	0.796	0.737
	Cross‐validation	0.8	0.8	0.845	0.791
	Validation	0.867	0.867	0.891	0.86
XGBoost	Training	1	1	1	1
	Cross‐validation	0.829	0.829	0.869	0.822
	Validation	0.967	0.967	0.969	0.967
LightGBM	Training	0.971	0.971	0.973	0.971
	Cross‐validation	0.9	0.9	0.916	0.901
	Validation	0.9	0.9	0.918	0.9
Naive Bayes	Training	0.9	0.9	0.902	0.9
	Cross‐validation	0.9	0.9	0.916	0.897
	Validation	0.933	0.933	0.933	0.933
Logistic regression (gradient descent method)	Training	0.614	0.614	0.655	0.593
	Cross‐validation	0.529	0.529	0.573	0.466
	Validation	0.533	0.533	0.522	0.487

*Note:* Accuracy: The proportion of correctly predicted samples out of the total samples. The higher the accuracy, the better. Recall rate (sensitivity or true positive rate): The proportion of actual positive samples that are correctly identified among all positive samples. The higher the recall, the better. Precision: the proportion of actual positive samples among all samples that are predicted as positive. The higher the precision, the better. F1 Score: the harmonic mean of precision and recall. Since precision and recall are often inversely related, the F1 score provides a balance between the two, especially when both are important.

### 3.6 Blood Metabolites HMDB0012085 and HMDB0009224 for Predicting Sprint Performance in Young Athletes

**FIGURE 8:**
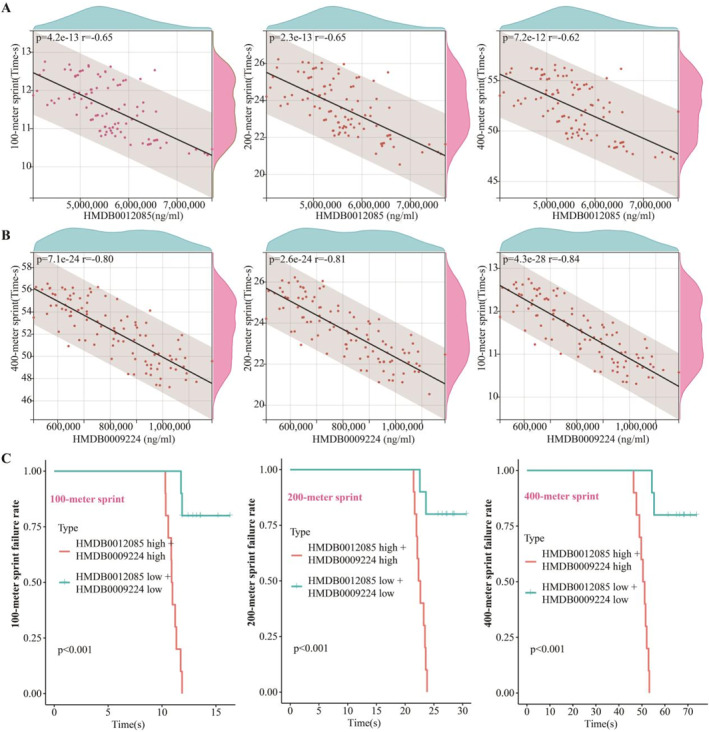
Elevated concentrations of the blood metabolites HMDB0012085 and HMDB0009224 exhibit a negative correlation with sprint performance times in healthy individuals and athletes. (A) Pearson's correlation analysis establishes a significant negative association between the blood levels of HMDB0012085 and the duration of physical activity. (B) Pearson's correlation analysis indicates a substantial negative relationship between the blood levels of HMDB0009224 and the length of exercise engaged in. (C) Kaplan–Meier analysis demonstrates that athletes with high expression levels of HMDB0012085 and HMDB0009224 have significantly lower failure rates in 100‐m, 200‐m, and 400‐m sprint events compared to those with low expression levels.

**FIGURE 9:**
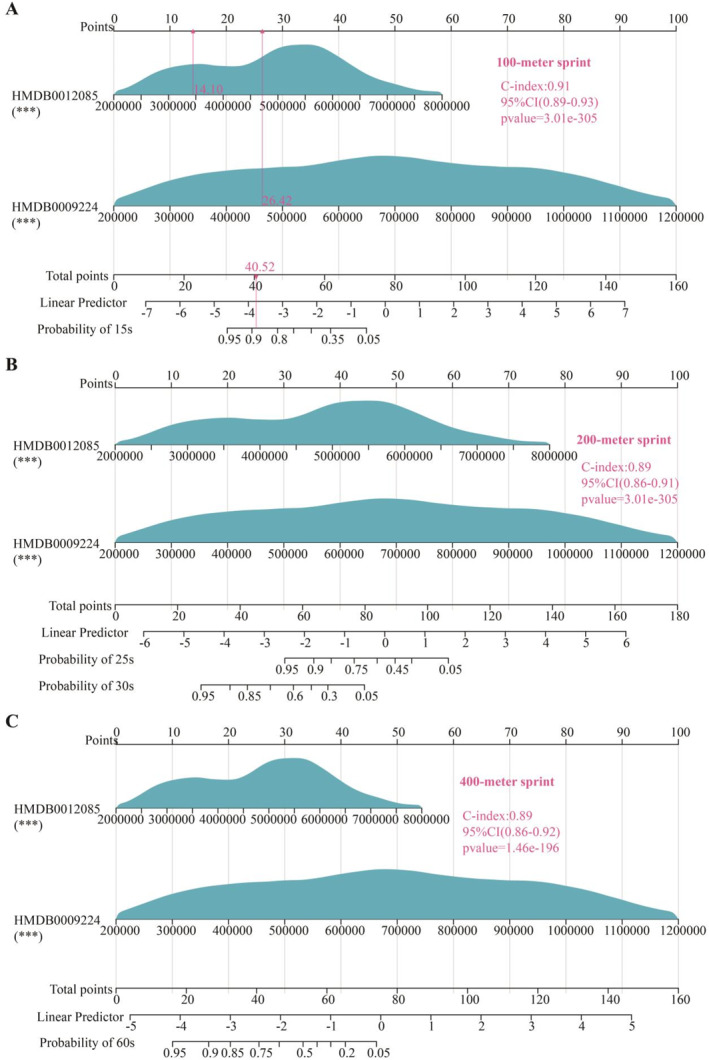
The nomogram analysis is utilized to assess the correlation between the levels of blood metabolites HMDB0012085 and HMDB0009224 and athletic performance in healthy individuals and athletes. (A–C) Among 100 athletes, the nomogram analysis results show that the levels of HMDB0012085 and HMDB0009224 are correlated with performance metrics in 100‐m (A), 200‐m (B), and 400‐m (C) sprint events. Scores include individual scores, denoted as “Point” in the figure, which represent the individual scores corresponding to different values of each variable, as well as the total score, denoted as “Total Point,” which represents the sum of the individual scores after all variables have taken their respective values. Predicted probability: For example, “Probability of 15s” in the figure indicates the probability of achieving a sprint time within 15 s.

**FIGURE 10:**
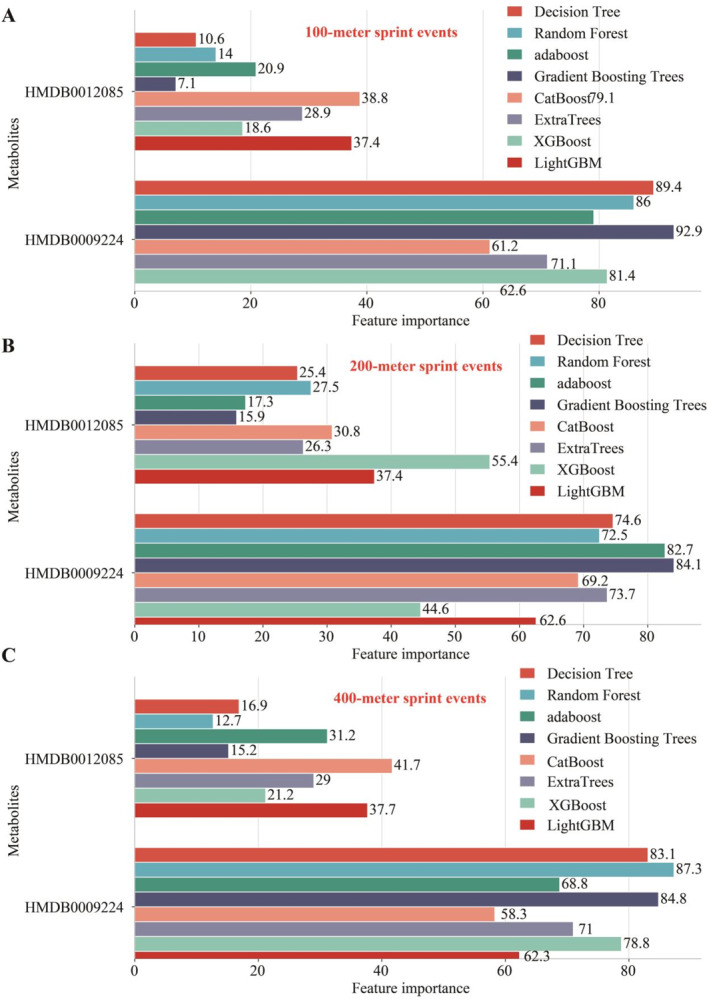
Feature importance of HMDB0012085 and HMDB0009224 in athletes' whole blood in relation to sports performance in athletes. (A) 100‐m sprint. (B) 200‐m sprint. (C) 400‐m sprint.

The concentrations of metabolites HMDB0012085 (Figure 8A) and HMDB0009224 (Figure 8B) in whole blood exhibit a significant negative correlation with sprint performance times. Kaplan–Meier analysis showed a substantial reduction in the incidence of suboptimal athletic performance among individuals with high expression levels of both metabolites, as opposed to those with low expression, across the 100‐m (Figure 8C), 200‐m (Figure 8D), and 400‐m (Figure 8E) sprint events. Additionally, nomogram analysis indicated that HMDB0012085 and HMDB0009224 are beneficial for assessing the athletic performance of both healthy individuals and young athletes in the 100‐m, 200‐m, and 400‐m sprint events (Figure 9A–C). Based on 12 types of machine learning algorithms, we assessed the efficacy of using the levels of HMDB0012085 and HMDB0009224 in the whole blood of athletes (*n* = 100) to predict 100‐m sprint performance (Table 2), 200‐m sprint performance (Table 3), and 400‐m sprint performance (Table 4). In the prediction the sprint performance of 100‐m sprinting (Figure 10A), 200‐m sprinting (Figure 10B), and 400‐m sprinting (Figure 10C), the feature importance of HMDB0009224 was superior to that of HMDB0012085.

**TABLE 2:** Machine learning models for blood metabolite profiling: Predict the sprint performance of young athletes using HMDB0012085 and HMDB0009224 in 100‐m sprint events.

Machine learning regression methods	Set	MSE	RMSE	MAE	MAPE	*R* ^2^
Decision tree	Training	0	0	0	0	1
	Cross‐validation	0.24	0.481	0.335	2.862	0.166
	Validation	0.164	0.405	0.262	2.177	0.657
Random forest	Training	0.013	0.113	0.084	0.722	0.968
	Cross‐validation	0.112	0.323	0.262	2.218	0.673
	Validation	0.141	0.375	0.313	2.71	0.712
Adaboost	Training	0	0.013	0.004	0.032	1
	Cross‐validation	0.271	0.505	0.394	3.376	0.42
	Validation	0.168	0.41	0.3	2.558	0.622
Gradient boosting trees	Training	0	0	0	0	1
	Cross‐validation	0.16	0.39	0.3	2.535	0.58
	Validation	0.351	0.592	0.43	3.622	0.144
CatBoost	Training	0	0.014	0.011	0.099	1
	Cross‐validation	0.168	0.394	0.319	2.744	0.466
	Validation	0.084	0.29	0.241	2.059	0.776
ExtraTrees	Training	0.014	0.117	0.091	0.78	0.97
	Cross‐validation	0.114	0.332	0.281	2.384	0.555
	Validation	0.085	0.291	0.235	2.037	0.837
K‐nearest neighbors	Training	0.127	0.356	0.31	2.665	0.736
	Cross‐validation	0.178	0.419	0.377	3.235	0.587
	Validation	0.121	0.348	0.308	2.65	0.751
Backpropagation neural network	Training	3.572	1.89	1.496	13.677	−7.596
	Cross‐validation	4.113	1.992	1.627	14.975	−15.628
	Validation	6.426	2.535	2.063	19.313	−9.746
Support vector machine	Training	1.093	1.045	0.867	7.296	−1.297
	Cross‐validation	0.597	0.674	0.56	4.598	−0.859
	Validation	1.547	1.244	1.048	8.678	−2.082
XGBoost	Training	0	0.007	0.005	0.041	1
	Cross‐validation	0.16	0.389	0.295	2.558	0.403
	Validation	0.187	0.432	0.344	2.926	0.61
LightGBM	Training	0.094	0.307	0.248	2.127	0.791
	Cross‐validation	0.211	0.455	0.393	3.395	−0.076
	Validation	0.1	0.316	0.265	2.221	0.745
Logistic regression (gradient descent method)	Training	0.077	0.277	0.216	1.856	0.834
	Cross‐validation	0.087	0.291	0.232	2.007	0.761
	Validation	0.097	0.311	0.248	2.072	0.79

*Note:* MSE (mean squared error): The expected value of the squared difference between the predicted and actual values. The smaller the value, the higher the model's accuracy. RMSE (root mean squared error): The square root of MSE. A smaller value indicates higher model accuracy. MAE (mean absolute error): The average of absolute errors, reflecting the actual situation of prediction errors. A lower value suggests better model accuracy. MAPE (mean absolute percentage error): A percentage‐based transformation of MAE. The lower the value, the higher the model's accuracy. *R* ^2^(coefficient of determination): Compares the prediction results with a model that uses only the mean value. The closer the result is to 1, the higher the model's accuracy.

**TABLE 3:** Machine learning models for blood metabolite profiling: Predict the sprint performance of young athletes using HMDB0012085 and HMDB0009224 in 200‐m sprint events.

Machine learning regression methods	Set	MSE	RMSE	MAE	MAPE	*R* ^2^
Decision tree	Training	0	0	0	0	1
	Cross‐validation	1.13	1.036	0.899	3.783	0.333
	Validation	1.42	1.191	0.934	3.952	0.325
Random forest	Training	0.095	0.309	0.262	1.105	0.953
	Cross‐validation	0.668	0.786	0.697	2.932	0.611
	Validation	0.527	0.726	0.609	2.509	0.75
Adaboost	Training	0.004	0.065	0.019	0.074	0.998
	Cross‐validation	0.639	0.783	0.631	2.646	0.489
	Validation	0.82	0.905	0.707	2.927	0.572
Gradient boosting trees	Training	0	0	0	0	1
	Cross‐validation	1.042	1	0.796	3.281	0.21
	Validation	0.503	0.709	0.554	2.365	0.77
CatBoost	Training	0.001	0.028	0.023	0.097	1
	Cross‐validation	0.609	0.773	0.61	2.514	0.655
	Validation	0.66	0.812	0.654	2.76	0.688
ExtraTrees	Training	0.08	0.283	0.214	0.901	0.963
	Cross‐validation	0.602	0.764	0.592	2.489	0.69
	Validation	0.4	0.633	0.571	2.36	0.75
K‐nearest neighbors	Training	0.562	0.749	0.617	2.573	0.742
	Cross‐validation	0.837	0.91	0.778	3.253	0.567
	Validation	0.621	0.788	0.622	2.608	0.683
Backpropagation neural network	Training	20.973	4.58	3.589	16.08	−8.47
	Cross‐validation	24.946	4.93	3.93	17.719	−14.198
	Validation	12.706	3.565	2.904	13.541	−6.043
Support vector machine	Training	147.807	12.158	9.122	73.649	−77.415
	Cross‐validation	30.528	4.29	3.43	25.397	−17.379
	Validation	131.632	11.473	9.595	10,864.137	−48.797
XGBoost	Training	0	0.018	0.011	0.047	1
	Cross‐validation	0.685	0.813	0.655	2.762	0.525
	Validation	0.51	0.714	0.507	2.061	0.788
LightGBM	Training	0.326	0.571	0.47	1.958	0.851
	Cross‐validation	0.67	0.807	0.683	2.862	0.599
	Validation	0.671	0.819	0.576	2.344	0.654
Logistic regression (gradient descent method)	Training	0.36	0.6	0.478	1.993	0.828
	Cross‐validation	0.445	0.635	0.521	2.164	0.777
	Validation	0.509	0.713	0.563	2.346	0.763

**TABLE 4:** Machine learning models for blood metabolite profiling: Predict the sprint performance of young athletes using HMDB0012085 and HMDB0009224 in 400‐m sprint events.

Machine learning regression methods	Set	MSE	RMSE	MAE	MAPE	*R* ^2^
Decision tree	Training	0	0	0	0	1
	Cross‐validation	5.381	2.285	1.725	3.275	−0.403
	Validation	3.236	1.799	1.178	2.185	0.646
Random forest	Training	0.459	0.678	0.506	0.966	0.938
	Cross‐validation	3.151	1.726	1.321	2.513	0.517
	Validation	1.74	1.319	0.945	1.754	0.543
Adaboost	Training	0.004	0.065	0.023	0.044	0.999
	Cross‐validation	3.133	1.734	1.339	2.526	0.371
	Validation	3.179	1.783	1.357	2.59	0.572
Gradient boosting trees	Training	0	0	0	0	1
	Cross‐validation	4.127	1.831	1.51	2.915	−0.049
	Validation	2.163	1.471	1.133	2.117	0.478
CatBoost	Training	0.005	0.073	0.058	0.109	0.999
	Cross‐validation	3.095	1.706	1.439	2.727	0.325
	Validation	3.564	1.888	1.395	2.666	0.557
ExtraTrees	Training	0.316	0.562	0.422	0.798	0.956
	Cross‐validation	2.522	1.54	1.241	2.332	0.519
	Validation	2.641	1.625	1.278	2.461	0.387
K‐nearest neighbors	Training	1.911	1.382	1.187	2.28	0.654
	Cross‐validation	2.669	1.61	1.409	2.713	0.371
	Validation	3.811	1.952	1.707	3.27	0.476
Backpropagation neural network	Training	85.914	9.269	7.485	15.151	−13.024
	Cross‐validation	104.607	9.795	8.176	16.603	−29.426
	Validation	62.494	7.905	6.146	12.759	−8.342
Support vector machine	Training	68.663	8.286	6.374	12.494	−8.892
	Cross‐validation	147.055	9.551	8.209	21.207	−27.618
	Validation	73.54	8.576	7.267	15.963	−15.979
XGBoost	Training	0.002	0.044	0.026	0.048	1
	Cross‐validation	4.637	2.052	1.693	3.236	0.184
	Validation	3.066	1.751	1.33	2.505	0.543
LightGBM	Training	1.262	1.123	0.962	1.816	0.767
	Cross‐validation	2.195	1.45	1.233	2.323	0.261
	Validation	4.184	2.045	1.637	3.155	0.415
Logistic regression (gradient descent method)	Training	1.929	1.389	1.142	2.188	0.65
	Cross‐validation	2.376	1.484	1.263	2.422	0.371
	Validation	1.662	1.289	1.134	2.157	0.786

## 4 Discussion

During the course of this research, we integrated a comprehensive set of analytical techniques—spanning from nontarget to targeted metabolomics and leveraging the power of machine learning—to uncover two critical insights. Firstly, we pinpointed the metabolites HMDB0012085 and HMDB0009224, which are notably elevated in athletes' blood compared to that of healthy individuals, effectively serving as discriminative markers between athletes and nonathletes. Secondly, within our sample of athletes, a robust negative correlation was detected between the levels of these metabolites and their sprint performance. Additionally, HMDB0012085 and HMDB0009224 have proven to be valuable predictors for the sprint times of athletes in the 100, 200, and 400 m events.

Nutritional substances are essential for sustaining and boosting endurance in sports. Athletes can markedly enhance their physical performance and stamina through a judicious diet rich in carbohydrates, proteins, fats, vitamins, and minerals. Carbohydrates are the main energy source during physical activities, especially in high‐intensity or endurance exercises, as they rapidly convert to glucose to fuel muscle function (Jeukendrup 2010). Proteins contribute to the repair and construction of muscle tissues which is crucial after strenuous workouts that can lead to muscle fiber damage (Moore 2021). Fats serve as a long‐term energy source, particularly important during extended, low‐intensity engagements (Volek, Noakes, and Phinney 2015). Vitamins and minerals participate in a myriad of physiological processes, such as metabolic energy conversion, muscle contraction, and nerve signal transmission, all of which can have an indirect impact on endurance (Peeling, Sim, and McKay 2023). Our research endeavors to identify blood metabolite biomarkers indicative of athletic potential, with the goal of elevating athletic performance through a well‐rounded diet and evidence‐based nutritional strategies.

The connection between exercise and metabolites is crucial as they jointly shape the body's response to physical activity. During exercise, to meet the increased energy demand, the body accelerates the breakdown of metabolites such as glucose, fatty acids, and amino acids to generate ATP, the energy molecule (Hargreaves et al. 2020; Grevendonk et al. 2021; Jaguri, Al Thani, and Elrayess 2023). Metabolites like lactic acid and creatine in the muscles are essential for muscle function and athletic performance, whereas the oxidation of fatty acids aids in weight management and endurance enhancement (Chávez‐Guevara et al. 2023). Exercise also affects hormone levels and their metabolites, thereby regulating energy balance and the ability to cope with stress. After exercise, the levels of metabolites related to inflammation and tissue repair rise within the body, and changes in neurotransmitter metabolites help improve mood and mental health (Egan et al. 2013). Long‐term adherence to exercise can promote adaptive changes in metabolic pathways, thereby enhancing athletic ability and overall health status. Therefore, metabolites play a key role in energy supply, muscle function, hormone regulation, mood impact, body temperature regulation, oxidative stress balance, metabolic pathway adjustment, and health adaptation (Hawley et al. 2014). In this study, we observed significant differences in the metabolite expression profiles in whole blood between healthy individuals (nonathletes) and athletes. This finding suggests that specific metabolites may be closely related to athletic potential, offering new biomarkers for understanding exercise capacity and providing potential metabolic targets for sports training and health maintenance.

In our study, we identified core DEMs, HMDB0012085, and HMDB0009224 which were significantly more abundant in athletes' whole blood compared to healthy individuals. According to the HMDB database, HMDB0012085, recognized as sphingomyelin (d18:0/14:0), is a key sphingolipid in animal cell membranes. Additionally, HMDB0009224, known as PE(20:0/18:1(9Z)), is a phosphatidylethanolamine (PE or GPEtn), a glycerophospholipid with a phosphorylethanolamine group. Our findings suggest that these metabolites may serve as biomarkers of physical performance capacity, reflecting the body's adaptability and proficiency in athletic activities. However, the expression levels of metabolites HMDB0012085 and HMDB0009224 were sometimes higher in controls than in athletes. This observed variability likely reflects genuine biological differences due to factors such as individual metabolic rates, diet, physical activity levels, or other environmental influences. These factors contribute to heterogeneity within each group, complicating the identification of robust biomarkers. To address this complexity, future studies should incorporate larger sample sizes to better capture the true distribution of metabolite levels across both groups. Larger cohorts will provide more robust data, enhancing biomarker discovery and validation. Additionally, integrating multi‐omics approaches and employing rigorous statistical methods can further improve the reliability and specificity of identified biomarkers.

Sphingomyelin's role in cell membranes, particularly in neurons and muscle fibers, is well‐established for its contribution to membrane stability and fluidity (Spector et al. 1985). The implications of sphingomyelin for athletic capacity extend beyond structural support, with emerging evidence linking it to sports performance through its involvement in cell signaling pathways that affect muscle contraction and energy metabolism (Hannun et al. 2018). The metabolites of sphingomyelin, including ceramide and sphingosine, are integral to cellular communication and stress response, potentially modulating muscle cell adaptation to exercise and, by extension, an individual's athletic prowess (Holthuis et al. 2001; Sezgin et al. 2017). Phosphatidylethanolamine, another critical phospholipid, is pervasive in cell membranes and contributes to cellular structure and function (Vance 2015). Its presence correlates directly with membrane fluidity and elasticity, essential for muscle cell function during physical activity. The fluidity of the cell membrane is a determinant of cellular responsiveness to stimuli, affecting muscle contraction efficiency and sports performance (van der Veen et al. 2017). Phosphatidylethanolamine also regulates intracellular calcium ion levels which are vital for muscle contraction thereby enhancing athletic potential (Knowles et al. 1975). The metabolic byproducts of phosphatidylethanolamine, such as diacylglycerol and triacylglycerol, are significant in energy metabolism, serving as energy reserves that can be rapidly mobilized during exercise to support muscle activity and endurance (Kawanishi et al. 2018; Gaspar et al. 2023). Our results indicate that HMDB0012085 and HMDB0009224 can effectively distinguish between healthy individuals and athletes, with HMDB0012085 showing superior feature importance for this differentiation. Moreover, these metabolites are effective in predicting sprint performance among athletes with HMDB0009224 demonstrating better predictive power for this specific athletic outcome.

By monitoring the levels of specific metabolites (HMDB0012085 and HMDB0009224), we can gain valuable insights into an athlete's physical condition and determine if they are primed for peak performance. Supplementation with nutrients that enrich these metabolites could potentially augment athletic capacity. Additionally, observing changes in these metabolite levels during varied training sessions can guide the development of personalized and more effective training programs, ensuring that athletes achieve their best possible outcomes.

However, our study has limitations, including being a single‐center trial with a limited sample size. Future multicenter studies with larger cohorts are needed to validate these findings and establish threshold values for HMDB0012085 and HMDB0009224. Additionally, our study did not include other indicators of athletic potential, which should be considered in future research for a more comprehensive assessment. Furthermore, The formulation of strategies for augmenting the levels of HMDB0012085 and HMDB0009224 necessitates a nuanced understanding of nutritional science. This approach should encompass considerations of optimal dietary sources, the bioavailability of these metabolites, and their integration into metabolic processes. Moreover, the potential applicability of HMDB0012085 and HMDB0009224 to a spectrum of sports disciplines merits rigorous evaluation. The efficacy of these metabolites in enhancing performance may be sport‐specific, thereby necessitating a tailored nutritional strategy for each athletic context. Comprehensive studies are essential to explore their role in various sports and to establish evidence‐based supplementation protocols. Lastly, our blood samples were collected 30 min before competition; further studies at various time points are required to confirm the optimal timing for precompetition interventions.

In summary, our study suggests that elevated blood levels of HMDB0012085 and HMDB0009224 may be biomarkers for distinguishing athletes from healthy individuals and could potentially predict athletic performance in terms of sprint events. These findings could aid in the selection and training of athletes.

## Ethics Statement

The study was carried out with the written informed consent of all participants, and with the approval of the Institutional Review Board of the Exercise and Health Sciences Department of Loughborough University, ensuring ethical standards were strictly adhered to throughout the research process.

## Conflicts of Interest

The authors declare no conflicts of interest.

## Supporting information

Supporting Information S1

Supporting Information S2

Supporting Information S3

Figure S1

Figure S2

Table S1

Table S2

## Data Availability

Data available on request from the corresponding author.

## References

[ejsc12272-bib-0001] Amendolara, A. , D. Pfister , M. Settelmayer , et al. 2023. “An Overview of Machine Learning Applications in Sports Injury Prediction.” Cureus 15: e46170. 10.7759/cureus.46170. 37905265 PMC10613321

[ejsc12272-bib-0002] Bonney, N. , P. Larkin , and K. Ball . 2022. “Kick Proficiency and Skill Adaptability Increase From an Australian Football Small‐Sided Game Intervention.” Frontiers in Sports and Active Living 4: 1026935. 10.3389/fspor.2022.1026935. 36385779 PMC9643701

[ejsc12272-bib-0003] Chávez‐Guevara, I. A. , F. J. Amaro‐Gahete , A. Ramos‐Jiménez , and J. F. Brun . 2023. “Toward Exercise Guidelines for Optimizing Fat Oxidation During Exercise in Obesity: A Systematic Review and Meta‐Regression.” Sports Medicine 53, no. 12: 2399–2416. 10.1007/s40279-023-01897-y. 37584843

[ejsc12272-bib-0004] Clarke, H. , Do‐H. Kim , C. A. Meza , M. J. Ormsbee , and R. C. Hickner . 2020. “The Evolving Applications of Creatine Supplementation: Could Creatine Improve Vascular Health?” Nutrients 12, no. 9: 2834. 10.3390/nu12092834. 32947909 PMC7551337

[ejsc12272-bib-0005] Egan, B. , and J. R. Zierath . 2013. “Exercise Metabolism and the Molecular Regulation of Skeletal Muscle Adaptation.” Cell Metabolism 17, no. 2: 162–184. 10.1016/j.cmet.2012.12.012. 23395166

[ejsc12272-bib-0006] Ferrando, A. A. , R. R. Wolfe , K. R. Hirsch , et al. 2023. “International Society of Sports Nutrition Position Stand: Effects of Essential Amino Acid Supplementation on Exercise and Performance.” Journal of the International Society of Sports Nutrition 20, no. 1: 2263409. 10.1080/15502783.2023.2263409. 37800468 PMC10561576

[ejsc12272-bib-0007] Fu, B. , and X.X. Fu . 2022. “Distributed Simulation System for Athletes' Mental Health in the Internet of Things Environment.” Computational Intelligence and Neuroscience 2022: 9186656–9186659. 10.1155/2022/9186656. 35371209 PMC8975696

[ejsc12272-bib-0008] Gaspar, R. C. , K. Lyu , B. T. Hubbard , et al. 2023. “Distinct Subcellular Localisation of Intramyocellular Lipids and Reduced PKCepsilon/PKCtheta Activity Preserve Muscle Insulin Sensitivity in Exercise‐Trained Mice.” Diabetologia 66, no. 3: 567–578. 10.1007/s00125-022-05838-8. 36456864 PMC11194860

[ejsc12272-bib-0009] Grevendonk, L. , N. J. Connell , C. McCrum , et al. 2021. “Impact of Aging and Exercise on Skeletal Muscle Mitochondrial Capacity, Energy Metabolism, and Physical Function.” Nature Communications 12, no. 1: 4773. 10.1038/s41467-021-24956-2. PMC8346468 34362885

[ejsc12272-bib-0010] Hannun, Y. A. , and L. M. Obeid . 2018. “Sphingolipids and Their Metabolism in Physiology and Disease.” Nature Reviews Molecular Cell Biology 19, no. 3: 175–191. 10.1038/nrm.2017.107. 29165427 PMC5902181

[ejsc12272-bib-0011] Hargreaves, M. , and L. L. Spriet . 2020. “Skeletal Muscle Energy Metabolism During Exercise.” Nature Metabolism 2, no. 9: 817–828. 10.1038/s42255-020-0251-4. 32747792

[ejsc12272-bib-0012] Hawley, J. A. , M. Hargreaves , M. J. Joyner , and J. R. Zierath . 2014. “Integrative Biology of Exercise.” Cell 159, no. 4: 738–749. 10.1016/j.cell.2014.10.029. 25417152

[ejsc12272-bib-0013] Holthuis, J. C. M. , T. Pomorski , R. J. Raggers , H. Sprong , and G. Van Meer . 2001. “The Organizing Potential of Sphingolipids in Intracellular Membrane Transport.” Physiological Reviews 81, no. 4: 1689–1723. 10.1152/physrev.2001.81.4.1689. 11581500

[ejsc12272-bib-0014] Jäger, R. , A. E. Mohr , K. C. Carpenter , et al. 2019. “International Society of Sports Nutrition Position Stand: Probiotics.” Journal of the International Society of Sports Nutrition 16, no. 1: 62. 10.1186/s12970-019-0329-0. 31864419 PMC6925426

[ejsc12272-bib-0015] Jaguri, A. , A. A. Al Thani , and M. A. Elrayess . 2023. “Exercise Metabolome: Insights for Health and Performance.” Metabolites 13, no. 6: 694. 10.3390/metabo13060694. 37367852 PMC10305288

[ejsc12272-bib-0016] James, C. , I. Jones , and S. Farra . 2022. “Physiological and Performance Correlates of Squash Physical Performance.” Journal of Sports Science and Medicine 21: 82–90. 10.52082/jssm.2022.82. 35250337 PMC8851109

[ejsc12272-bib-0017] Jeukendrup, A. E. 2010. “Carbohydrate and Exercise Performance: The Role of Multiple Transportable Carbohydrates.” Current Opinion in Clinical Nutrition and Metabolic Care 13, no. 4: 452–457. 10.1097/mco.0b013e328339de9f. 20574242

[ejsc12272-bib-0018] Kawanishi, N. , K. Takagi , H.‐C. Lee , et al. 2018. “Endurance Exercise Training and High‐Fat Diet Differentially Affect Composition of Diacylglycerol Molecular Species in Rat Skeletal Muscle.” American Journal of Physiology – Regulatory, Integrative and Comparative Physiology 314, no. 6: R892–R901. 10.1152/ajpregu.00371.2017. 29443549 PMC6032301

[ejsc12272-bib-0019] Knowles, A. F. , A. Kandrach , E. Racker , and H. G. Khorana . 1975. “Acetyl Phosphatidylethanolamine in the Reconstitution of Ion Pumps.” Journal of Biological Chemistry 250, no. 5: 1809–1813. 10.1016/s0021-9258(19)41766-3. 122978

[ejsc12272-bib-0020] Lee, S. , Y. Choi , E. Jeong , et al. 2023. “Physiological Significance of Elevated Levels of Lactate by Exercise Training in the Brain and Body.” Journal of Bioscience and Bioengineering 135, no. 3: 167–175. 10.1016/j.jbiosc.2022.12.001. 36681523

[ejsc12272-bib-0021] Moore, D. R. 2021. “Protein Requirements for Master Athletes: Just Older Versions of Their Younger Selves.” Sports Medicine 51, no. S1: 13–30. 10.1007/s40279-021-01510-0. 34515969 PMC8566396

[ejsc12272-bib-0022] Peeling, P. , M. Sim , and A. K. A. McKay . 2023. “Considerations for the Consumption of Vitamin and Mineral Supplements in Athlete Populations.” Sports Medicine 53, no. S1: 15–24. 10.1007/s40279-023-01875-4. 37358750 PMC10721676

[ejsc12272-bib-0023] Reis, F. J. J. , R. K. Alaiti , C. S. Vallio , and L. Hespanhol . 2024. “Artificial Intelligence and Machine Learning Approaches in Sports: Concepts, Applications, Challenges, and Future Perspectives.” Brazilian Journal of Physical Therapy 28, no. 3: 101083. 10.1016/j.bjpt.2024.101083. 38838418 PMC11215955

[ejsc12272-bib-0024] Semenova, E. A. , E. C. R. Hall , and I. I. Ahmetov . 2023. “Genes and Athletic Performance: The 2023 Update.” Genes 14, no. 6: 1235. 10.3390/genes14061235. 37372415 PMC10298527

[ejsc12272-bib-0025] Sezgin, E. , I. Levental , S. Mayor , and C. Eggeling . 2017. “The Mystery of Membrane Organization: Composition, Regulation and Roles of Lipid Rafts.” Nature Reviews Molecular Cell Biology 18, no. 6: 361–374. 10.1038/nrm.2017.16. 28356571 PMC5500228

[ejsc12272-bib-0026] Spector, A. A. , and M. A. Yorek . 1985. “Membrane Lipid Composition and Cellular Function.” Journal of Lipid Research 26, no. 9: 1015–1035. 10.1016/s0022-2275(20)34276-0. 3906008

[ejsc12272-bib-0027] Taber, C. B. , S. Sharma , M. S. Raval , et al. 2024. “A Holistic Approach to Performance Prediction in Collegiate Athletics: Player, Team, and Conference Perspectives.” Scientific Reports 14, no. 1: 1162. 10.1038/s41598-024-51658-8. 38216641 PMC10786827

[ejsc12272-bib-0028] Vance, J. E. 2015. “Phospholipid Synthesis and Transport in Mammalian Cells.” Traffic 16: 1–18. 10.1111/tra.12230. 25243850

[ejsc12272-bib-0029] van der Veen, J. N. , J. P. Kennelly , S. Wan , J. E. Vance , D. E. Vance , and R. L. Jacobs . 2017. “The Critical Role of Phosphatidylcholine and Phosphatidylethanolamine Metabolism in Health and Disease.” Biochimica et Biophysica Acta (BBA) – Biomembranes 1859, no. 9: 1558–1572. 10.1016/j.bbamem.2017.04.006. 28411170

[ejsc12272-bib-0030] Volek, J. S. , T. Noakes , and S. D. Phinney . 2015. “Rethinking Fat as a Fuel for Endurance Exercise.” European Journal of Sport Science 15, no. 1: 13–20. 10.1080/17461391.2014.959564. 25275931

[ejsc12272-bib-0031] Wilkerson, M. D. , and D. N. Hayes . 2010. “ConsensusClusterPlus: A Class Discovery Tool With Confidence Assessments and Item Tracking.” Bioinformatics 26, no. 12: 1572–1573. 10.1093/bioinformatics/btq170. 20427518 PMC2881355

