# Supplementary material for: Predicting Sprint Potential: A Machine Learning Model Based on Blood Metabolite Profiles in Young Male Athletes
Source: Eur J Sport Sci. 2025 Feb 24;25(3):e12272. doi: 10.1002/ejsc.12272 (PMC11849406; doi:10.1002/ejsc.12272)
Supplement: Supplementary file 2 — Supporting Information S2 [file EJSC-25-e12272-s007.docx]

**Supplementary Figure 1. UMAP plots of all metabolite profiles in healthy and athlete groups.** UMAP plots displaying the distribution of all metabolites in the healthy group (blue) and athlete group (red). Each point represents an individual sample, illustrating the overall metabolic profile.
