# Supplementary material for: Predicting Sprint Potential: A Machine Learning Model Based on Blood Metabolite Profiles in Young Male Athletes
Source: Eur J Sport Sci. 2025 Feb 24;25(3):e12272. doi: 10.1002/ejsc.12272 (PMC11849406; doi:10.1002/ejsc.12272)
Supplement: Supplementary file 3 — Supporting Information S3 [file EJSC-25-e12272-s005.docx]

**Supplemental Figure 2. Confusion matrix heatmap of binary classification model performance.** This figure presents a confusion matrix heatmap to evaluate the performance of our binary classification model. The heatmap provides a clear and concise visualization of the model's prediction accuracy, with each cell color intensity reflecting the frequency of occurrences. (A) Decision Trees, (B) Random Forest, (C) AdaBoost, (D) Gradient Boosting Trees, (E) CatBoost, (F) ExtraTrees, (G)K-Nearest Neighbors, (H) Backpropagation Neural Networks, (I) Support Vector Machines, (J) XGBoost, (K) LightGBM, (L) Naive Bayes, and (M) Logistic Regression employing the Gradient Descent method.
