# Supplementary figures and images for: Predicting Sprint Potential: A Machine Learning Model Based on Blood Metabolite Profiles in Young Male Athletes
Source: Eur J Sport Sci. 2025 Feb 24;25(3):e12272. doi: 10.1002/ejsc.12272 (PMC11849406; doi:10.1002/ejsc.12272)

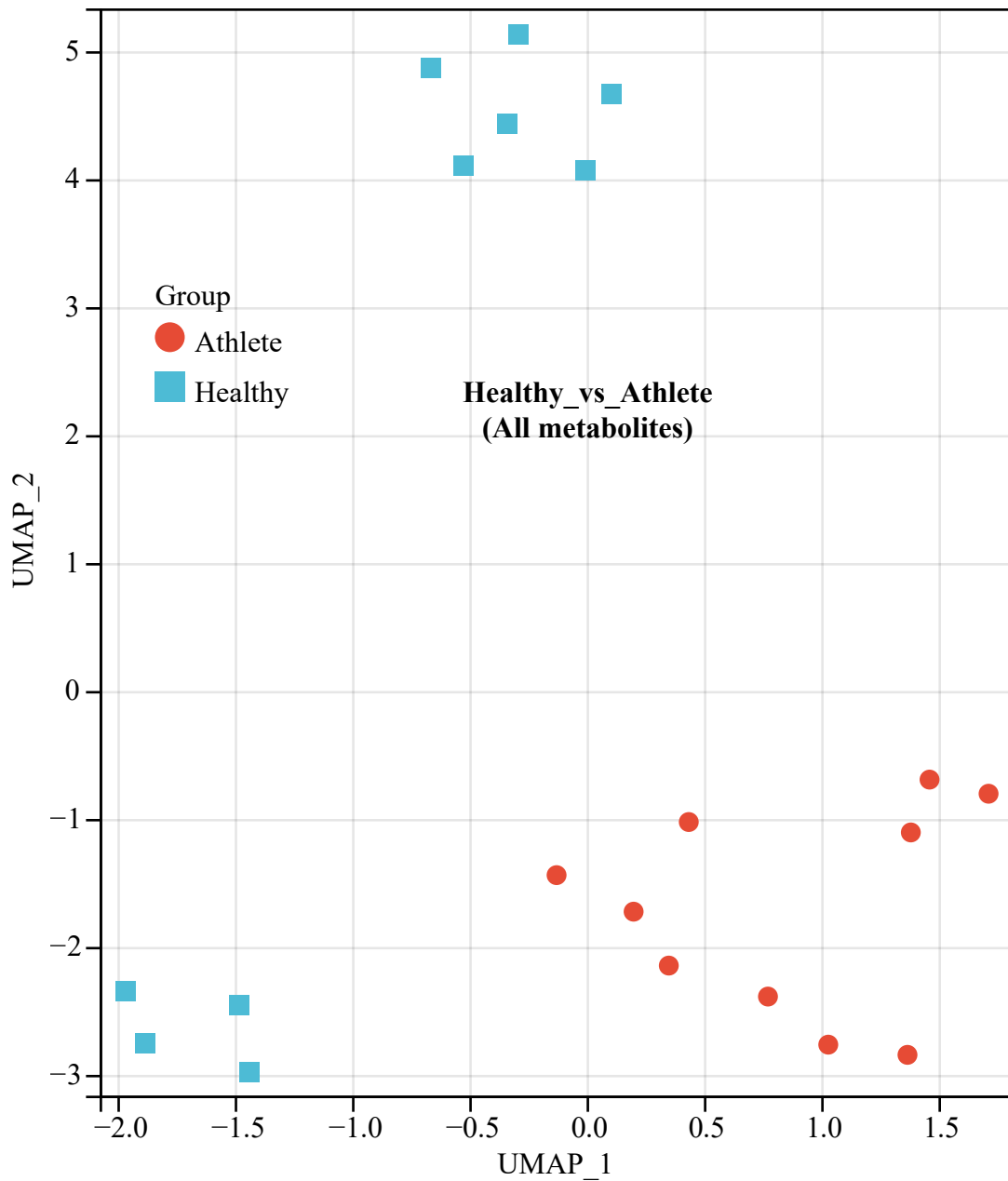

Supplement: Supplementary file 4 — Figure S1 [file EJSC-25-e12272-s003.pdf]

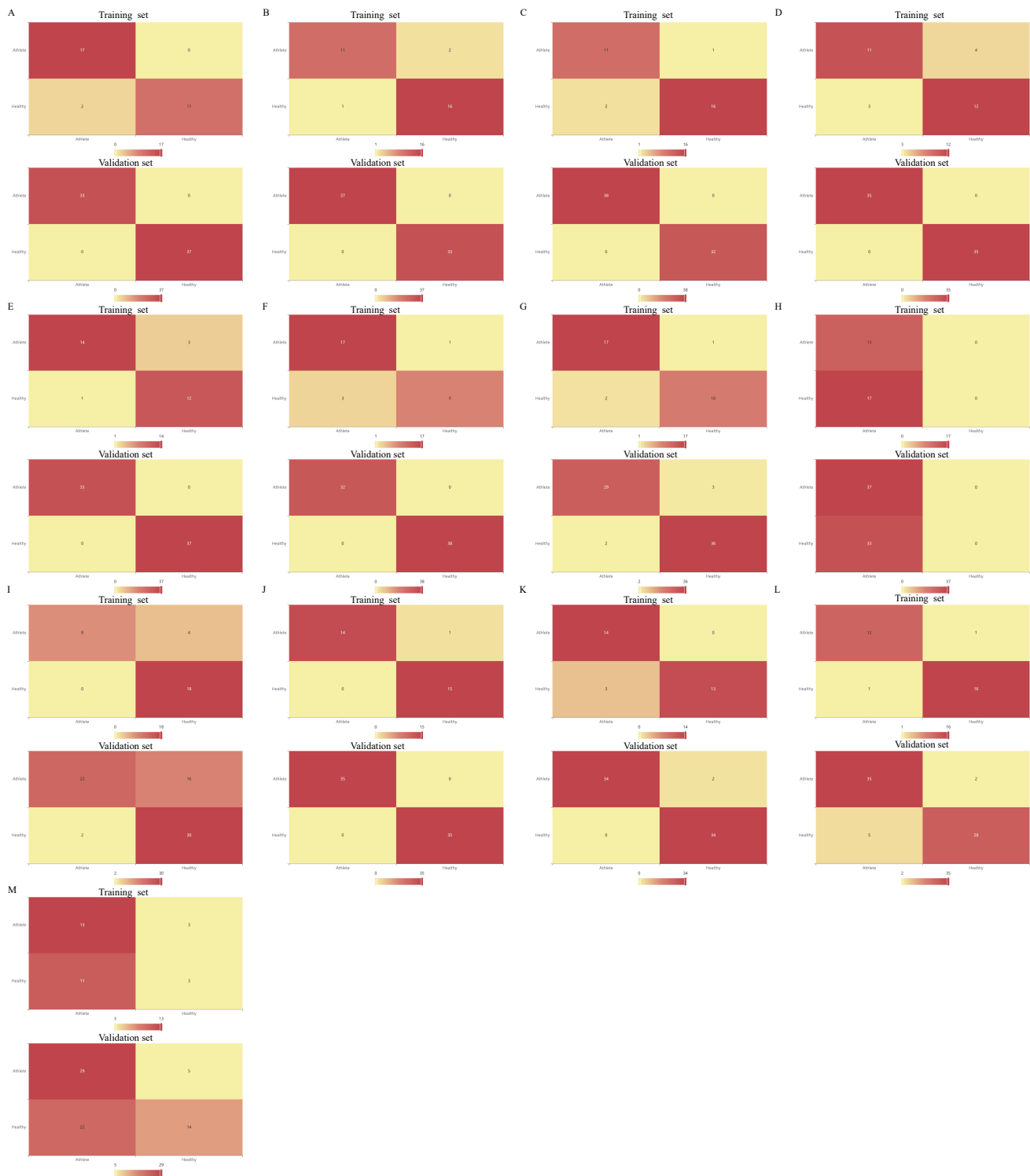

Supplement: Supplementary file 5 — Figure S2 [file EJSC-25-e12272-s001.pdf]
