# Supplementary material for: Predicting Sprint Potential: A Machine Learning Model Based on Blood Metabolite Profiles in Young Male Athletes
Source: Eur J Sport Sci. 2025 Feb 24;25(3):e12272. doi: 10.1002/ejsc.12272 (PMC11849406; doi:10.1002/ejsc.12272)
Supplement: Supplementary file 6 — Table S1 [file EJSC-25-e12272-s004.doc]

**Supplementary table 1. The detailed description of the parameters for each machine learning model used in this study.**

| **Methods** | **Items** | **Parameter value** |
| --- | --- | --- |
| Decision Tree | Data segmentation | 0.7 |
|  | Data Cleaning | Yes |
|  | Cross verification | 5 |
|  | Evaluation criteria for node splitting | gini |
|  | Feature division point selection criteria | best |
|  | The maximum proportion of features considered when dividing | None |
|  | Minimum number of samples of internal node splits | 2 |
|  | Minimum sample number of leaf nodes | 1 |
|  | The minimum weight of samples in leaf nodes | 0 |
|  | Maximum number of leaf nodes | 50 |
|  | The maximum depth of the tree | 10 |
|  | The threshold value of node partition impurity | 0 |
| Random Forest | Data segmentation | 0.7 |
|  | Data Cleaning | Yes |
|  | Cross verification | 5 |
|  | Evaluation criteria for node splitting | gini |
|  | Decision tree quantity | 100 |
|  | Sampling with replacement | TRUE |
|  | Out-of-bag testing | FALSE |
|  | The maximum proportion of features considered when dividing | auto |
|  | Minimum number of samples of internal node splits | 2 |
|  | Minimum sample number of leaf nodes | 1 |
|  | The minimum weight of samples in leaf nodes | 0 |
|  | The maximum depth of the tree | 10 |
|  | Maximum number of leaf nodes | 50 |
|  | The threshold value of node partition impurity | 0 |
| adaboost | Data segmentation | 0.7 |
|  | Data Cleaning | Yes |
|  | Cross verification | 5 |
|  | Number of base classifiers | 100 |
|  | Learning rate | 1 |
| Gradient Boosting Trees | Data segmentation | 0.7 |
|  | Data Cleaning | Yes |
|  | Cross verification | 5 |
|  | Loss function | deviance |
|  | Evaluation criteria for node splitting | friedman_mse |
|  | Number of base learners | 100 |
|  | Learning rate | 0.1 |
|  | Sampling without replacement ratio | 1 |
|  | The maximum proportion of features considered when dividing | None |
|  | Minimum number of samples of internal node splits | 2 |
|  | Minimum sample number of leaf nodes | 1 |
|  | The minimum weight of samples in leaf nodes | 0 |
|  | The maximum depth of the tree | 10 |
|  | Maximum number of leaf nodes | 50 |
|  | Impurity threshold for node splitting | 0 |
| CatBoost | Data segmentation | 0.7 |
|  | Data Cleaning | Yes |
|  | Cross verification | 5 |
|  | Number of iterations | 100 |
|  | Learning rate | 0.1 |
|  | L2 regularization | 1 |
|  | The maximum depth of the tree | 10 |
|  | Overfitting detection threshold | 0 |
|  | Post-convergence iterations | 20 |
| ExtraTrees | Data segmentation | 0.7 |
|  | Data Cleaning | Yes |
|  | Cross verification | 5 |
|  | The maximum proportion of features considered when dividing | None |
|  | Minimum number of samples of internal node splits | 2 |
|  | Minimum sample number of leaf nodes | 1 |
|  | The minimum weight of samples in leaf nodes | 0 |
|  | The maximum depth of the tree | 10 |
|  | Maximum number of leaf nodes | 50 |
|  | Maximum number of leaf nodes | 50 |
|  | The threshold value of node partition impurity | 0 |
| K-Nearest Neighbors | Data segmentation | 0.7 |
|  | Data Cleaning | Yes |
|  | Cross verification | 5 |
|  | Search algorithm | auto |
|  | Number of leaves | 30 |
|  | Near neighbor number | 5 |
|  | Nearest neighbor sample weight function | uniform |
|  | Vector distance algorithm | euclidean |
| Backpropagation Neural Network | Data segmentation | 0.7 |
|  | Data Cleaning | Yes |
|  | Cross verification | 5 |
|  | Activation function | identity |
|  | Solver | lbfgs |
|  | Learning rate | 0.1 |
|  | L2 regularization | 1 |
|  | Number of iterations | 1000 |
|  | Hide the number of layer 1 neurons | 100 |
| Support Vector Machine | Data segmentation | 0.7 |
|  | Data Cleaning | Yes |
|  | Cross verification | 5 |
|  | Penalty coefficient | 1 |
|  | Kernel function | linear |
|  | Kernel function coefficient | scale |
|  | Kernel function constant | 0 |
|  | The highest degree of the kernel function | 3 |
|  | Error convergence | 0.001 |
|  | Maximum number | 1000 |
|  | Multi-class fusion | ovr |
| XGBoost | Data segmentation | 0.7 |
|  | Data Cleaning | Yes |
|  | Cross verification | 5 |
|  | Base learner | gbtree |
|  | Number of base learners | 100 |
|  | Learning rate | 0.1 |
|  | L1 regularization | 0 |
|  | L2 regularization | 1 |
|  | Sample sampling rate | 1 |
|  | Tree feature sampling rate | 1 |
|  | Node feature sampling rate | 1 |
|  | The minimum weight of samples in leaf nodes | 0 |
|  | The maximum depth of the tree | 10 |
| LightGBM | Data segmentation | 0.7 |
|  | Data Cleaning | Yes |
|  | Cross verification | 5 |
|  | Base learner | gbdt |
|  | Number of base learners | 100 |
|  | Learning rate | 0.1 |
|  | L1 regularization | 0 |
|  | L2 regularization | 1 |
|  | Sample sampling rate | 1 |
|  | Tree feature sampling rate | 1 |
|  | Node split threshold | 0 |
|  | The minimum weight of samples in leaf nodes | 0 |
|  | The maximum depth of the tree | 10 |
|  | Minimum sample number of leaf nodes | 10 |
| Logistic Regression (Gradient Descent Method) | Data segmentation | 0.7 |
|  | Data Cleaning | Yes |
|  | Cross verification | 5 |
|  | Regularization | none |
|  | Set the intercept | TRUE |
|  | Error convergence condition | 0.001 |
|  | Maximum Number of iterations | 1000 |
| Naive Bayes | Data segmentation | 0.7 |
|  | Data Cleaning | Yes |
|  | Cross verification | 5 |
|  | Prior distribution | Gaussian distribution |
|  | alpha | 1 |
|  | Binarization threshold | 0 |
