# Supplementary material for: Predicting Sprint Potential: A Machine Learning Model Based on Blood Metabolite Profiles in Young Male Athletes
Source: Eur J Sport Sci. 2025 Feb 24;25(3):e12272. doi: 10.1002/ejsc.12272 (PMC11849406; doi:10.1002/ejsc.12272)
Supplement: Supplementary file 7 — Table S2 [file EJSC-25-e12272-s006.doc]

**Supplementary Table 2. Functional analysis of 257 DEMs via MetaboAnalyst 6.0 database .**

| **HMDB** | **Name** | **log2**  **(fold change)** | **pvalue** | **Pathway** |
| --- | --- | --- | --- | --- |
| HMDB0000564 | PC(32:0) | -0.956334352 | 9.30294E-06 | Glycerophospholipid metabolism |
| HMDB0007906 | PC(14:1(9Z)/18:1(9Z)) | 1.305466103 | 1.38402E-06 | Glycerophospholipid metabolism |
| HMDB0007907 | PC(14:1(9Z)/18:2(9Z,12Z)) | 0.911585257 | 0.002100181 | Glycerophospholipid metabolism |
| HMDB0007912 | PC(14:1(9Z)/20:1(11Z)) | 1.169142509 | 0.000254318 | Glycerophospholipid metabolism |
| HMDB0007936 | PC(15:0/16:1(9Z)) | 0.692090174 | 0.033135833 | Glycerophospholipid metabolism |
| HMDB0007937 | PC(15:0/18:0) | -0.951104029 | 4.6675E-05 | Glycerophospholipid metabolism |
| HMDB0007951 | PC(15:0/20:5(5Z,8Z,11Z,14Z,17Z)) | 0.753465619 | 0.021514345 | Glycerophospholipid metabolism |
| HMDB0007969 | PC(16:0/16:1(9Z)) | -0.913718141 | 1.9349E-05 | Glycerophospholipid metabolism |
| HMDB0007982 | PC(16:0/20:4(5Z,8Z,11Z,14Z)) | -0.714154776 | 0.001361605 | Glycerophospholipid metabolism |
| HMDB0007984 | PC(16:0/20:5(5Z,8Z,11Z,14Z,17Z)) | -0.745932683 | 0.001420328 | Glycerophospholipid metabolism |
| HMDB0007985 | PC(16:0/22:0) | -0.677488 | 0.003468463 | Glycerophospholipid metabolism |
| HMDB0007989 | PC(38:5) | -0.815522352 | 0.000435311 | Glycerophospholipid metabolism |
| HMDB0008032 | PC(18:0/14:1(9Z)) | 1.322794507 | 1.14357E-06 | Glycerophospholipid metabolism |
| HMDB0008048 | PC(18:0/20:4(5Z,8Z,11Z,14Z)) | -0.953098172 | 4.06293E-05 | Glycerophospholipid metabolism |
| HMDB0008054 | PC(40:4) | -1.072335646 | 1.38917E-08 | Glycerophospholipid metabolism |
| HMDB0008055 | PC(40:5) | -0.996212563 | 3.80786E-06 | Glycerophospholipid metabolism |
| HMDB0008057 | PC(40:6) | -0.830168104 | 0.000193713 | Glycerophospholipid metabolism |
| HMDB0008077 | PC(18:1(11Z)/20:1(11Z)) | 0.675061894 | 0.029947128 | Glycerophospholipid metabolism |
| HMDB0008092 | PC(42:2) | -0.816087512 | 0.000731533 | Glycerophospholipid metabolism |
| HMDB0008114 | PC(18:1(9Z)/20:4(5Z,8Z,11Z,14Z)) | -1.001679383 | 1.42338E-05 | Glycerophospholipid metabolism |
| HMDB0008285 | PC(20:0/22:4(7Z,10Z,13Z,16Z)) | -0.600344203 | 0.025388782 | Glycerophospholipid metabolism |
| HMDB0008288 | PC(42:6) | -0.719166597 | 0.00718063 | Glycerophospholipid metabolism |
| HMDB0008307 | PC(20:1(11Z)/20:0) | -1.036542768 | 1.98886E-06 | Glycerophospholipid metabolism |
| HMDB0008321 | PC(20:1(11Z)/22:6(4Z,7Z,10Z,13Z,16Z,19Z)) | -0.594918493 | 0.019745768 | Glycerophospholipid metabolism |
| HMDB0008580 | PC(22:1(13Z)/22:4(7Z,10Z,13Z,16Z)) | -1.067689075 | 1.044E-07 | Glycerophospholipid metabolism |
| HMDB0008596 | PC(22:2(13Z,16Z)/18:1(11Z)) | -0.807986075 | 0.001795521 | Glycerophospholipid metabolism |
| HMDB0008626 | PC(22:4(7Z,10Z,13Z,16Z)/16:0) | -0.88354265 | 7.746E-05 | Glycerophospholipid metabolism |
| HMDB0008923 | PE(16:0/16:0) | -0.955750376 | 8.41823E-06 | Glycerophospholipid metabolism |
| HMDB0008925 | PE(16:0/18:0) | -0.829209422 | 0.000159916 | Glycerophospholipid metabolism |
| HMDB0008937 | PE(16:0/20:4(5Z,8Z,11Z,14Z)) | -0.896786833 | 0.000216407 | Glycerophospholipid metabolism |
| HMDB0008943 | PE(16:0/22:4(7Z,10Z,13Z,16Z)) | -1.083859878 | 1.97351E-06 | Glycerophospholipid metabolism |
| HMDB0008958 | PE(16:1(9Z)/18:0) | -0.621194328 | 0.010561928 | Glycerophospholipid metabolism |
| HMDB0008970 | PE(16:1(9Z)/20:4(5Z,8Z,11Z,14Z)) | -0.791411087 | 0.001764686 | Glycerophospholipid metabolism |
| HMDB0008976 | PE(16:1(9Z)/22:4(7Z,10Z,13Z,16Z)) | -0.788980519 | 0.000726663 | Glycerophospholipid metabolism |
| HMDB0009005 | PE(18:0/20:5(5Z,8Z,11Z,14Z,17Z)) | -0.800338582 | 0.001992748 | Glycerophospholipid metabolism |
| HMDB0009009 | PE(18:0/22:4(7Z,10Z,13Z,16Z)) | -0.865883259 | 0.00019288 | Glycerophospholipid metabolism |
| HMDB0009012 | PE(18:0/22:6(4Z,7Z,10Z,13Z,16Z,19Z)) | -0.695995476 | 0.004028679 | Glycerophospholipid metabolism |
| HMDB0009023 | PE(18:1(11Z)/16:1(9Z)) | -0.645093974 | 0.005821212 | Glycerophospholipid metabolism |
| HMDB0009036 | PE(18:1(11Z)/20:4(5Z,8Z,11Z,14Z)) | -0.878516649 | 6.7557E-05 | Glycerophospholipid metabolism |
| HMDB0009057 | PE(18:1(9Z)/18:0) | -0.807085252 | 0.0006028 | Glycerophospholipid metabolism |
| HMDB0009087 | PE(18:2(9Z,12Z)/15:0) | 0.762727911 | 0.018046543 | Glycerophospholipid metabolism |
| HMDB0009099 | PE(18:2(9Z,12Z)/20:2(11Z,14Z)) | 0.670201482 | 0.041375766 | Glycerophospholipid metabolism |
| HMDB0009115 | PE(18:2(9Z,12Z)/P-18:0) | 1.235446963 | 1.43888E-06 | Glycerophospholipid metabolism |
| HMDB0009224 | PE(20:0/18:1(9Z)) | -0.94064656 | 4.68218E-08 | Glycerophospholipid metabolism |
| HMDB0009288 | PE(20:2(11Z,14Z)/18:0) | -0.697933202 | 0.012129199 | Glycerophospholipid metabolism |
| HMDB0009302 | PE(20:2(11Z,14Z)/20:5(5Z,8Z,11Z,14Z,17Z)) | 0.856911411 | 0.005894745 | Glycerophospholipid metabolism |
| HMDB0009387 | PE(20:4(5Z,8Z,11Z,14Z)/18:0) | -1.072370952 | 6.09558E-09 | Glycerophospholipid metabolism |
| HMDB0009408 | PE(20:4(5Z,8Z,11Z,14Z)/22:6(4Z,7Z,10Z,13Z,16Z,19Z)) | -0.97269352 | 1.21195E-05 | Glycerophospholipid metabolism |
| HMDB0009527 | PE(22:1(13Z)/20:1(11Z)) | -0.813153831 | 0.000884911 | Glycerophospholipid metabolism |
| HMDB0009682 | PE(22:6(4Z,7Z,10Z,13Z,16Z,19Z)/16:0) | -0.675427828 | 0.005061232 | Glycerophospholipid metabolism |
| HMDB0009719 | PE(24:0/18:1(9Z)) | -0.630737079 | 0.002299607 | Glycerophospholipid metabolism |
| HMDB0007865 | PA(18:1(9Z)/18:1(9Z)) | -0.682664066 | 0.002664183 | Glycerophospholipid metabolism |
| HMDB0000895 | Acetylcholine | 0.72079135 | 0.03226512 | Glycerophospholipid metabolism |
| HMDB0010166 | PS(18:0/22:5(7Z,10Z,13Z,16Z,19Z)) | -0.656738383 | 0.009125449 | Glycerophospholipid metabolism |
| HMDB0010167 | PS(18:0/22:6(4Z,7Z,10Z,13Z,16Z,19Z)) | -0.654007062 | 0.009634774 | Glycerophospholipid metabolism |
| HMDB0010379 | LysoPC(14:0/0:0) | -0.72370429 | 0.004126495 | Glycerophospholipid metabolism |
| HMDB0010381 | LysoPC(15:0/0:0) | 0.838596161 | 0.008759621 | Glycerophospholipid metabolism |
| HMDB0010383 | LysoPC(16:1/0:0) | -0.674150107 | 0.006877142 | Glycerophospholipid metabolism |
| HMDB0010390 | LysoPC(20:0/0:0) | -1.060640262 | 4.15136E-07 | Glycerophospholipid metabolism |
| HMDB0010391 | LysoPC(20:1(11Z)/0:0) | -1.038010039 | 7.63606E-08 | Glycerophospholipid metabolism |
| HMDB0010399 | LysoPC(22:1(13Z)/0:0) | -0.670654619 | 0.010283522 | Glycerophospholipid metabolism |
| HMDB0001316 | 6-Phosphogluconic acid | -0.6325912 | 0.004037385 | Pentose phosphate pathway |
| HMDB0001351 | Deoxyribose 1-phosphate | -0.696432866 | 0.006113801 | Pentose phosphate pathway |
| HMDB0000163 | D-Maltose | 0.770035532 | 0.021919263 | Starch and sucrose metabolism |
| HMDB0000975 | Trehalose | 0.770035532 | 0.021919263 | Starch and sucrose metabolism |
